# MRS-BIDS, an extension to the Brain Imaging Data Structure for magnetic resonance spectroscopy

**DOI:** 10.1038/s41597-025-05543-2

**Published:** 2025-08-08

**Authors:** Amy E. Bouchard, Dickson Wong, Wolfgang Bogner, Rémi Gau, Yaroslav O. Halchenko, Damon G. Lamb, Christopher J. Markiewicz, Paul G. Mullins, Guiomar Niso, Georg Oeltzschner, BIDS Maintainers, Rémi Gau, Christopher J. Markiewicz, Stefan Appelhoff, Ross Blair, Eric Earl, Anthony Galassi, Nell Hardcastle, Julia-Katharina Pfarr, Kimberly Ray, Christine Rogers, Taylor Salo, William T. Clarke, Martin Wilson, Mark Mikkelsen

**Affiliations:** 1 Department of Radiology, Weill Cornell Medicine, New York, NY USA; 2 Department of Clinical Neurological Sciences, Schulich School of Medicine and Dentistry, Western University, London, ON Canada; 3 Comprehensive Center for AI in Medicine (CAIM), Medical University of Vienna, Vienna, Austria; 4 Christian Doppler Laboratory – BIOMAK, Medical University of Vienna, Vienna, Austria; 5 High-field MR Center, Department of Biomedical Imaging and Image-guided Therapy, Medical University of Vienna, Vienna, Austria; 6 Université Paris-Saclay, CEA, Inria, Gif-sur-Yvette, France; 7 Department of Psychological and Brain Sciences, Dartmouth College, Hanover, NH USA; 8 Departments of Psychiatry, Neuroscience, and Biomedical Engineering, University of Florida, Gainesville, FL USA; 9 Center for OCD, Anxiety, and Related Disorders, University of Florida, Gainesville, FL USA; 10 Center for Cognitive Aging and Memory, University of Florida, Gainesville, FL USA; 11 McKnight Brain Institute, University of Florida, Gainesville, FL USA; 12 Brain Rehabilitation Research Center, Malcom Randall Veterans Affairs Medical Center, Gainesville, FL USA; 13 Department of Psychology, Stanford University, Stanford, CA USA; 14 Bangor Imaging Unit, School of Psychology and Sport Science, Bangor University, Bangor, UK; 15 Instituto Cajal, CSIC, Madrid, Spain; 16 Russell H. Morgan Department of Radiology and Radiological Science, The Johns Hopkins University School of Medicine, Baltimore, MD USA; 17 F. M. Kirby Research Center for Functional Brain Imaging, Kennedy Krieger Institute, Baltimore, MD USA; 18 Wellcome Centre for Integrative Neuroimaging, FMRIB, Nuffield Department of Clinical Neurosciences, University of Oxford, Oxford, UK; 19 Centre for Human Brain Health and School of Psychology, University of Birmingham, Birmingham, UK; 20 Max Planck Institute for Human Development, Berlin, Germany; 21 Intramural Research Program, National Institute of Mental Health, Bethesda, MD USA; 22 Montreal Neurological Institute, McGill University, Montreal, QC Canada; 23 University of Texas, Austin, TX USA; 24 Department of Psychiatry, University of Pennsylvania, Philadelphia, PA USA; 25 Penn Lifespan Informatics and Neuroimaging Center (PennLINC), Philadelphia, PA USA; 26 Lifespan Brain Institute, Penn Medicine and Children’s Hospital of Philadelphia, Philadelphia, PA USA

**Keywords:** Neuroscience, Molecular neuroscience, Research management

## Abstract

The Brain Imaging Data Structure (BIDS) is an increasingly adopted standard for organizing scientific data and metadata. It facilitates easier and more straightforward data sharing and reuse. BIDS currently encompasses several biomedical imaging and non-imaging techniques, and as more research groups begin to use it, additional experimental techniques are being incorporated into the standard, allowing diverse experimental methods to be stored within the same cohesive structure. Here, we present an extension for magnetic resonance spectroscopy (MRS) data, termed MRS-BIDS.

## Introduction

Magnetic resonance spectroscopy (MRS), an application of the nuclear magnetic resonance phenomenon, for the discovery of which the 1944 Nobel Prize in Physics was awarded to Isidor Isaac Rabi, preceded the development of magnetic resonance imaging (MRI) by several decades. Its ability to noninvasively detect and estimate the concentration of endogenous chemical compounds *in vivo* has made it a powerful tool for studying metabolism in many contexts. Today, *in vivo* MRS is employed to interrogate the molecular underpinnings of many diseases and disorders, such as autism spectrum disorder^1^, cancer^2^, multiple sclerosis^3^, and schizophrenia^4^, to name a few. It is also used to study the neurochemical correlates of behavior, cognition, and perception, such as memory^5^, motor performance^6^, learning^7^, and vision^8^. However, a perennial issue with MRS, particularly compared to MRI, is the lack of standardization in terminology, acquisition approaches, data preprocessing, data analysis, metabolite quantification, and reporting of results. Recently, the MRS community came together to reach consensus recommendations for various aspects of MRS science. These recommendations were published as a series of articles in a special issue of *NMR in Biomedicine*^9^. In parallel, the Code and Data Sharing Committee of the MR Spectroscopy Study Group of the International Society for Magnetic Resonance in Medicine (ISMRM) was formed^10^. The Committee promotes the creation, curation, and sharing of openly available MRS datasets, software tools, educational materials, and expert knowledge for new and experienced users in the wider MRS community. A website was subsequently created to serve as a curated resource and hub for data, code, and resource sharing, as well as a virtual space for community interaction to discuss relevant topics of proposed and ongoing MRS research (https://mrshub.org/).

Owing to the large variety of MRS acquisition techniques, experimental designs, proprietary data formats, and analysis software toolkits, there are currently no widely accepted data and metadata management and organization practices. This has traditionally made collaborations and data exchange between (and even within) laboratories challenging, as adjusting workflows to different data structures can not only lead to a loss of time in retrieving data but also cause analytic or interpretation errors from a misunderstanding of the data or results, and ultimately failure to reproduce scientific conclusions from the same underlying data. The standardized data accumulation logic inherent to BIDS^11^ offers a generalizable organization framework and is a potential solution to research- and reproducibility-related limitations of non-standardized workflows. BIDS was initially established to share anatomical, functional, and diffusion-weighted MRI data through the OpenNeuro data repository^12^ and proposes a hierarchical organization of experimental data with different levels for unequivocal identification of each dataset (i.e., subject, session, and imaging/non-imaging modality). BIDS also requires a minimal set of metadata to perform adequate data processing and analyses. It has now been extended to include arterial spin labeling^13^, electroencephalography^14^, genetics^15^, intracranial electroencephalography^16^, magnetoencephalography^17^, microscopy^18^, motion^19^, near-infrared spectroscopy^20^, positron emission tomography^21^, and quantitative MRI^22^. BIDS conforms with the FAIR Guiding Principles^23^, namely, “findability”, “accessibility”, “interoperability”, and “reusability”. BIDS directly addresses all but accessibility, which itself is addressed by repositories that support BIDS datasets (e.g., OpenNeuro). Findability and reusability are achieved by storing crucial metadata in human- and machine-readable sidecar files, whereas interoperability is achieved using standardized data formats, such as the Neuroimaging Informatics Technology Initiative (NIfTI) file format. The current documentation describing BIDS can be found online (https://bids-specification.readthedocs.io/en/stable/).

To further expand BIDS, this article presents an extension to the specification for MRS data and metadata. Much of the community discussion around the extension proposal was conducted online via Google Docs and refined through GitHub. Note that the MRS-BIDS has now been fully incorporated into the overarching BIDS specification.

## MRS-BIDS Summary

As with all BIDS datasets, MRS-BIDS datasets comply with the parent BIDS nomenclature and data and metadata organization structure. While in this extension we have adopted MRS terminology described in a recent consensus paper^24^, we also conform to the extent possible with Digital Imaging and Communications in Medicine (DICOM) terminology to match the current BIDS philosophy. While some labels and fields must be included to comply with the BIDS standard (e.g., a label to uniquely identify a subject or MRS-specific metadata like spectral width), the majority are optional or strongly encouraged.

**Fig. 1:**
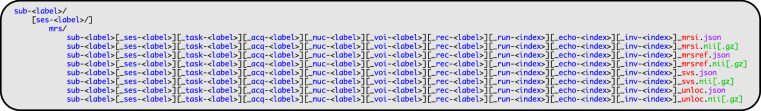
Template of MRS data and metadata file organization in a BIDS dataset directory with emphasis on the MRS-BIDS specification. If entities (in blue) are in square brackets, they are optional to use and should be used only when users need to distinguish between files in the same directory; otherwise, the entity is required. Every MRS data file ends in a suffix (in red) that indicates the data file type, and every NIfTI-MRS data file (in green) has a corresponding JSON sidecar (in purple).

BIDS follows well-defined file naming conventions and a directory/subdirectory hierarchy as illustrated in Fig. 1. Filenames consist of zero or more entities, one suffix, and an extension: <entities>_<suffix>.<extension>. Entities are key-value pairs, such as sub-<label>, where label is an alphanumeric value. Users are generally free to choose their own labels, but they must be used consistently throughout the entire dataset. The suffix indicates the data type of the file. MRS can encode spectral signals from a single volume (single-voxel spectroscopy, SVS) or along 1, 2, or 3 spatial dimensions, resulting in multiple sub-volumes (MRS imaging, MRSI). For SVS and MRSI data, users must use the suffixes svs and mrsi, respectively, to differentiate them from each other. If localization was not used, the suffix unloc must be used. Additionally, some researchers commonly collect an additional MRS acquisition used as a reference for preprocessing or for scaling metabolite signal levels, such as the signal from an external reference (e.g., a phantom) or internal tissue water. For these datasets, the suffix mrsref must be used. Finally, the extension indicates the file format, such as nii[.gz] for a compressed NIfTI-1 or NIfTI-2 file.

BIDS files are contained in a directory structure of the form sub-<label>/[ses-<label>]/<datatype>/, where the mandatory sub-<label> and optional ses-<label> directories match the entities found in the filenames, and the <datatype>/ subdirectories separate distinct recording methodologies, such as anat/ for anatomical images and eeg/ for electroencephalographic recordings. MRS-specific data files must be stored in a data type subdirectory named mrs/. Moreover, in this extension, we introduce the entities nuc-<label> and voi-<label> to indicate, respectively, the target nucleus (e.g., nuc-1H, for proton; nuc-31P, for phosphorus; or nuc-1H13C, for dual-tuned proton–carbon acquisitions) and volume of interest (VOI) (e.g., voi-acc or voi-pcc for data collected in the anterior or posterior cingulate cortices). As a reminder, labels can be user-defined. Additionally, we adopt the preexisting BIDS entities: task-<label>, acq-<label>, rec-<label>, run-<index>, echo-<index>, and inv-<index>. These entities indicate the name of the task the subject underwent, acquisition parameters, reconstruction algorithms, run number, echo time, and inversion time of the acquisition. The acq label is quite useful as it allows users to differentiate files with differences in acquisition parameters that have a substantial impact on the data collected. For example, when multiple signal references are collected for the same subject, in the same session, one for metabolite concentration referencing and the other for eddy-current correction, the files could be named sub-01_acq-concref_mrsref.nii.gz and sub-01_acq-ecc_mrsref.nii.gz. In the context of MRS, rec-<label> could be used to distinguish signal sampling algorithms (e.g., in MRSI experiments, users might implement various EPI reconstruction approaches, such as those that use spectral prior knowledge or spatial spectral knowledge). The run-<index> key-value pair is used when an acquisition is repeated in the same session (e.g., in a test-retest experiment). echo-<index> and inv-<index> are used to indicate parametric acquisitions, or “entity-linked file collections”. That is, two or more files that differ only by echo and/or inversion time(s) are considered part of the same acquisition. Note that <index> must be a nonnegative integer; for echo and inv, it does not refer to the actual echo or inversion time.

Putting this all together, a proton (^1^H) MRS scan in a BIDS dataset from subject 6’s second scan session that involved an *n*-back memory task collected from a single VOI in the dorsolateral prefrontal cortex using the semi-LASER sequence could be named: sub-06_ses-02_task-nback_acq-slaser_nuc-1H_voi-dlpfc_svs.nii.gz. Note that entities task, acq, nuc, and voi would be required only if they were needed to differentiate between other acquisitions in this subject’s second session subdirectory of MRS data files; otherwise, they are optional. That said, users should avoid creating unnecessarily lengthy filenames; the minimum requirement is that filenames contain enough information to make them distinct. Filenames must begin with the string sub-<label>_ses-<label>, or just sub-<label> if ses-<label> is not used. Additionally, filenames must end with a suffix and a file extension, _<suffix>.<extension>. All other entities between the subject/session and suffix/extension entities have a fixed order, as shown in Fig. 1, and must be used only once in a filename.

## Specific MRS-BIDS Considerations

**Fig. 2:**
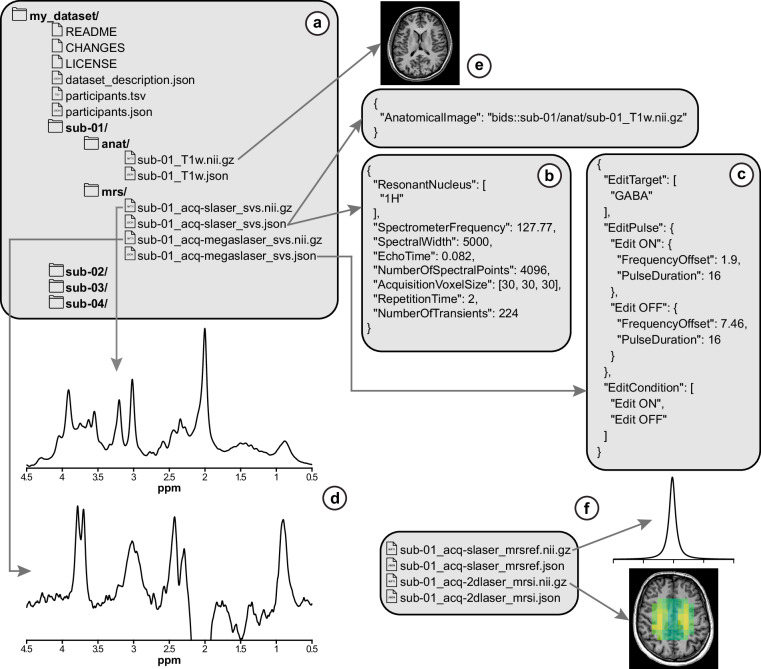
Example directory and file structure of MRS data and metadata using MRS-BIDS. (**a**) The BIDS file system hierarchy with the mrs/ directory included. (**b**) Contents of the NIfTI-MRS file JSON sidecar. The required key-value pairs and some recommended ones are shown. (**c**) These key-value pairs would be included in the sidecar for spectral-edited MRS data. (**d**) Example sLASER and MEGA-sLASER spectra that would be stored in the NIfTI-MRS data files. (**e**) If an anatomical image is collected during a study, "AnatomicalImage" should be included in the JSON sidecar. (**f**) Additional NIFTI-MRS dataset examples, one of an unsuppressed water reference signal and one of 2D MRSI, and how the data files would be named.

Each major MRI manufacturer exports MRS data in one or more proprietary file formats, making it challenging for software developers to design their applications to handle all the possible data formats users may produce. Although there is a DICOM storage class capable of storing raw MRS data, manufacturer support is inconsistent, and custom research sequence support requires the development of inline reconstruction pipelines. Thus, a significant hurdle to the standardization of MRS methodology is attributable to this diversity. As a complementary initiative to this BIDS extension, we have developed an open-source file format for MRS data based on the NIfTI standard, termed NIfTI-MRS^25,26^ (https://wtclarke.github.io/mrs_nifti_standard/). A NIfTI-MRS file contains a standard NIfTI header and a seven-dimensional data block where all raw data and metadata can be easily stored and parsed. In addition, the NIfTI-MRS header has an embedded JavaScript Object Notation (JSON) extension where BIDS-compliant metadata can be stored (JSON sidecar files are described in a later section). It is important to state that to comply with MRS-BIDS, all raw data must be converted into NIfTI-MRS format for easier interoperability across software tools. Such conversion can be performed using the Python-based spec2nii program (https://github.com/wtclarke/spec2nii). Figure 2 illustrates how a typical MRS study consisting of NIfTI-MRS and JSON files would be organized according to MRS-BIDS principles. A full and detailed description of BIDS file structures and hierarchies, and the contents of JSON files, can be found online: https://bids-specification.readthedocs.io/en/stable/. The modality-specific definitions for MRS may be found in the following subsection of the full standard: https://bids-specification.readthedocs.io/en/stable/modality-specific-files/magnetic-resonance-spectroscopy.html.

Since it is ideal to (re)analyze data with little or no preprocessing already applied to it, the “rawness” of the data stored in the NIfTI-MRS file is contingent upon the format of the source file. Therefore, users should export their MRS data in a format that is as “raw” as possible based on their MR scanner’s capabilities before conversion. For MRSI data, “raw” means data that have already been spatially reconstructed (i.e., from (*k*,*t*)-space to image space) to avoid the complexities of sampling approaches. The NIfTI-MRS format is not designed to store MRSI data that has not been spatially reconstructed. If desired, users may store their unreconstructed MRSI data in the sourcedata directory (https://bids-specification.readthedocs.io/en/stable/common-principles.html#source-vs-raw-vs-derived-data).

Moreover, it is commonplace in MRS studies to acquire high-resolution 3D anatomical MR images for accurate voxel or acquisition slab placement, generation of co-registered voxel masks, and tissue segmentation for voxel-based partial-volume tissue correction. If an anatomical image was collected, we recommend users include the field "AnatomicalImage" in the NIfTI-MRS JSON sidecar file. This field contains the relative path(s) to the corresponding anatomical MR image file(s) as a string or array of strings.

It is helpful to provide additional details regarding acquisition parameters to differentiate datasets since a great variety of MRS sequences exist. As such, several optional labels based on the most popular MRS sequences/techniques can be used when using acq-<label> in the filename. Researchers ultimately can choose any label they want if it stays consistent across participants and sessions and uses allowable label characters. If this entity is used, the chosen label should be described in the "PulseSequenceType" field in the accompanying JSON file. A selection of suggested labels includes press, steam, laser, slaser, special, mega, hermes, hercules, mqc (multiple quantum coherence editing), lcosy (localized correlation spectroscopy), j (*j*-resolved spectroscopy), dw (diffusion-weighted spectroscopy), fid, mc (metabolite-cycled spectroscopy), and spinecho. It should be noted that these labels may be combined if needed to describe the acquisition (e.g., jpress).

Furthermore, the nuc-<label> entity can be used to differentiate acquisitions tuned to detect different nuclei. If nuc-<label> is used, then the field "ResonantNucleus" must be included in the accompanying JSON file using the label as the value entered. In addition, the voi-<label> entity can be used to differentiate acquisitions with different VOI. Using voi-<label> requires that the "BodyPart" and "BodyPartDetails" fields also be present in the JSON sidecar file. "BodyPartDetailsOntology" is optional.

## JSON Sidecar Files

BIDS prescribes that all data files have a JSON sidecar file that details the metadata related to the accompanying data. Several categories of JSON metadata fields, such as those related to scanner hardware, sequence specifics, tissue description, and MRS-relevant fields, can be included. MRS-BIDS shares many of the same key-value pairs in the JSON sidecar as the other MR modalities. For brevity, only those that were introduced in this extension or are especially relevant to MRS are described. All other key-value pairs can be found on the BIDS specification website (see link above).

### Scanner hardware

Optional fields include "NumberReceiveCoilActiveElements" and "NumberTransmitCoilActiveElements", which refer to the number of active elements used by the receive and transmit radiofrequency (RF) coils, respectively.

### Sequence specifics

Concerning sequence specifics, recommended fields include "WaterSuppression", a Boolean that indicates whether water suppression was employed before the data were acquired and must be either true or false. Other optional fields include "WaterSuppressionTechnique", "OuterVolumeSuppression", "B0ShimmingTechnique", and "B1ShimmingTechnique". "WaterSuppressionTechnique" is the name of the applied water suppression technique. "OuterVolumeSuppression" is a Boolean stating whether outer-volume suppression was employed before the data were collected and must be true or false. "B0ShimmingTechnique" and "B1ShimmingTechnique" describe how the *B*_0_ and *B*_1_ fields were shimmed, respectively.

### Tissue description

For a description of the tissue that is sampled, optional files include "BodyPart", "BodyPartDetails", and "BodyPartDetailsOntology". If the voi-<label> entity is used in the data filename, "BodyPart" and "BodyPartDetails" must be included in the JSON file; "BodyPartDetailsOntology" is optional. More specifically, "BodyPart" refers to the scanned body region or part of the scanned organ; "BodyPartDetails" outlines extra details about the body part or location; and "BodyPartDetailsOntology" details a Uniform Resource Identifier (URI) used for "BodyPartDetails" (see, e.g., https://www.ebi.ac.uk/ols4/ontologies/uberon/).

### MRS-relevant fields

Some MRS-relevant fields, such as "ResonantNucleus", "SpectrometerFrequency", "SpectralWidth", and "EchoTime", must be included. First, "ResonantNucleus" refers to the isotope of interest of the MRS experiment. Second, "SpectrometerFrequency" reflects the spectrometer frequency (in MHz). Third, "SpectralWidth" reports the spectral bandwidth of the sampled MR signal (in Hz). Fourth, "EchoTime" reports the echo time of the acquisition (in s).

Other recommended fields are "NumberOfSpectralPoints", "MixingTime", "AcquisitionVoxelSize", "ReferenceSignal", "NumberOfTransients", "MatrixSize", "VolumeAffineMatrix","EncodingTechnique", and "AnatomicalImage". First, "NumberOfSpectralPoints" is the number of complex data points in each recorded transient of the time-domain MRS signal, equal to the number of points in one spectrum. Second, for a stimulated-echo MRS sequence, "MixingTime" is the interval between stimulated-echo pulses (in s). Third, "AcquisitionVoxelSize" corresponds to the nominal voxel size or voxel resolution of a VOI or acquisition slab and is an array of numbers having a length of 3 (in mm). Fourth, "ReferenceSignal" details the water-unsuppressed reference file(s) path(s) to the corresponding svs, mrsi, or unloc NIfTI-MRS data file, if applicable. Furthermore, "NumberOfTransients" corresponds to how many times one application of the pulse sequence is recorded for an MRS acquisition. Note that this is recommended for SVS and unlocalized acquisitions only. Additionally, "MatrixSize" is an array of integers with a length of 3, corresponding to the acquisition slab’s matrix size. "VolumeAffineMatrix" is an array of arrays specifying a 4 × 4 matrix (with the same conventions and coordinates as the NIfTI *{qs}form* affine matrix) representing the orientation, position, and size of an additional VOI. That is, this matrix defines a VOI in addition to the primary method of localization (e.g., PRESS localization) that allows for unambiguous identification of any RF-localized volume separate from the field of view. Further, "EncodingTechnique" corresponds to the encoding technique used during the readout. It should be noted that "MatrixSize", "VolumeAffineMatrix", and "EncodingTechnique" are all recommended for MRSI. Lastly, "AnatomicalImage" refers to the path(s) to the anatomical MR image file(s) for the corresponding MRS data file.

"ChemicalShiftOffset", "ChemicalShiftReference", "EditTarget", "EditPulse", "EditCondition", "EchoAcquisition", "PulseSequenceTiming", "PulseSequencePulses", and "ReceiveGain" are also additional optional fields. "ChemicalShiftOffset" is the chemical shift (in ppm) in the middle of "SpectralWidth", analogous to 0 Hz. "ChemicalShiftReference" denotes the chemical shift at the transmitter frequency (in ppm). "EditTarget" describes the targeted metabolites, "EditPulse" details the editing parameters if spectral editing was used, and "EditCondition" details the application order of "EditPulse". "EchoAcquisition" details the way in which the detected echo was recorded while the analog-to-digital converter was turned on. "PulseSequenceTiming" details the time (in s) when each RF pulse of the acquisition pulse sequence was played out in relation to the start of the acquisition (i.e., from echo time = 0 ms). Relatedly, "PulseSequencePulses" is a list of the pulses that were applied. If included in the JSON sidecar, the array size of "PulseSequencePulses" must equal the array size of "PulseSequenceTiming". Lastly, "ReceiveGain" denotes the receive gain of the receive coil.

## Example MRS-BIDS Datasets

Several examples of MRS-BIDS-compliant datasets can be found on the BIDS GitHub repository site (https://github.com/bids-standard/bids-examples) or other open-access repositories. The data files are publicly available, but the NIfTI files are empty. Complete repositories can be found on Zenodo and NITRC:

- *2D semi-LASER MRSI reproducibility dataset* [bids-examples/mrs_2dmrsi/] (10.5281/zenodo.7189139)^27^. These data were collected from 8 subjects as part of the SLIPMAT pipeline to extract tissue-specific spectral profiles from MRSI data^28^.
- *Big GABA*: The dataset located at https://github.com/bids-standard/bids-examples/tree/master/mrs_biggaba is a BIDS-compliant subset of a larger repository at https://www.nitrc.org/projects/biggaba^29^. The latter comprises data collected for a large multi-vendor, multi-site project to study the measurement outcomes of GABA-edited MRS^30^. The dataset found in the BIDS examples repository is a subset of this larger dataset, consisting of 12 subjects collected from one site.
- *fMRS in pain*: The dataset located at https://github.com/bids-standard/bids-examples/tree/master/mrs_fmrs is a BIDS-compliant version of the dataset at https://www.nitrc.org/projects/fmrs_2020^31^. This is a set of functional MRS datasets collected from 15 subjects to study neurochemical responses to pain stimuli^32^.

## MRS Software Packages Compliant with BIDS

Developers of several MRS software packages are beginning to program their toolkits to be fully BIDS compliant. Osprey^33^ and Gannet^34^ can read in NIfTI-MRS data files, but are not yet able to parse a fully BIDS-organized dataset automatically. Additionally, the spant^35^ R package offers some functionality for writing and reading BIDS datasets, while FSL-MRS^36^ supports BIDS-formatted directories through third-party software.

## Future Modifications to MRS-BIDS

When modeling MRS spectral data, it is typical to utilize prior knowledge. For spectral fitting, this is done using a basis set of metabolite signal resonances created by quantum mechanical numerical simulations. This forms an essential part of the analysis and quantification pipeline. MRS-BIDS currently does not have specifications for storing basis set data and metadata. Future updates to MRS-BIDS should consider addressing this as a matter of priority. For instance, it is unclear whether basis sets should be considered raw or derivative data.

## Conclusions

The motivation for creating the MRS-BIDS extension to the BIDS specification stemmed from a desire for the same degree of standardization that is common for other biomedical imaging and non-imaging modalities and, fundamentally, to adhere to the principles of FAIR. As the need for big data repositories continues to grow, adopting a data and metadata organization standard is essential, and the BIDS extension we have developed ensures MRS data follows suit. Modern science and research are clearly shifting toward greater standardization and openness in methodology and results. MRS-BIDS signifies a valuable advancement for MRS science in this regard, and it is hoped that the broader MRS community will embrace it.
